# American ginseng (*Panax quinquefolius*) administration does not affect performance of the Roche COBAS Ampliprep/Taqman HIV-1 RNA assay

**DOI:** 10.1186/1472-6882-14-427

**Published:** 2014-10-31

**Authors:** Rahul P Bakshi, Todd T Brown, Antoine Simmons, Chun-Su Yuan, Brent A Bauer, Jeff A Sloan, Adriana Andrade

**Affiliations:** Department of Medicine, Division of Clinical Pharmacology, Johns Hopkins University, Osler 503, 600 N Wolfe Street, Baltimore, MD 21287 USA; Department of Medicine, Division of Endocrinology, Diabetes, and Metabolism, Johns Hopkins University, 1830 East Monument Street, Suite 333, Baltimore, MD 21287 USA; Department of Pathology, Johns Hopkins University, 600 North Wolfe Street Meyer Bldg, Rm B130, Baltimore, MD 21287 USA; Department of Anesthesia and Critical Care, Tang Center for Herbal Medicine Research, Department of Anesthesia & Critical Care Pritzker School of Medicine, University of Chicago, 5841 S. Maryland Avenue, MC 4028, Chicago, Il 60637 USA; Department of Medicine, Mayo Clinic, 200 1st St SW, Rochester, MN 55905 USA; Department of Health Sciences Research, Mayo Clinic, Harwick 8-29, 200 First Street SW, Rochester, MN 55905 USA; Division of Infectious Diseases, Johns Hopkins University, 1830 E. Monument Street, Suite 8074, Baltimore, MD 21205 USA

## Abstract

**Background:**

Previous data indicate that purified components of ginseng can inhibit HIV reverse transcriptase *in vitro*, suggesting that ginseng components in plasma may interfere with HIV-1 RNA detection assays.

**Methods:**

Pre- and post-dose plasma from three volunteers dosed with 3000 mg American ginseng was spiked with HIV and analyzed by the Roche COBAS Ampliprep/Taqman v2.0 HIV-1 RNA assay.

**Results:**

Presence of American ginseng had no significant effect on measured HIV-1 RNA concentration. Variation within pre- and post-dose plasma pair was insignificant and within assay performance limits.

**Conclusion:**

Plasma from subjects dosed with 3000 mg American ginseng does not interfere with the Roche COBAS Ampliprep/Taqman v2.0 HIV-1 RNA assay. This implies that *in vitro* inhibition of HIV reverse transcriptase by American ginseng components is unlikely to be clinically relevant.

## Background

American ginseng is used to ameliorate HIV-associated fatigue [1]. Recent reports have indicated that protein components of Asian ginseng (*Panax ginseng* and *Panax notoginseng*) can inhibit HIV-1 reverse transcriptase and other polymerases *in vitro* [2, 3]. Data from the literature suggests that it is unlikely that protein components of orally-dosed ginseng are bioavailable at relevant concentrations, or survive the processing required to quantify plasma HIV-1 RNA concentrations [4, 5]. However, these findings raised concerns that ginseng components may interfere with reverse transcriptase-dependent assays used to determine plasma HIV-1 RNA concentration, and potentially confound clinical management of this disease.

We hypothesized that standardized American ginseng would not affect the performance of the Roche COBAS Ampliprep/Taqman v2.0 HIV-1 RNA assay (Limit of Detection 20 copies/ml) used in clinical practice to quantify plasma HIV-1 RNA. American ginseng was selected for this experiment because we used this dietary supplement in our previous studies [6, 7]. We tested our hypothesis by measuring the performance of the Roche COBAS Ampliprep/Taqman v2.0 HIV-1 RNA assay [4] in plasma of volunteers receiving high-dose American ginseng.

## Methods

De-identified archived plasma samples from healthy volunteers and HIV-infected patients were utilized with Johns Hopkins Medicine Institutional Review Board approval. Test samples had been archived from a previous study (NCT01136928, http://www.clinicaltrials.gov) in healthy volunteers dosed orally with 3000 mg daily of American ginseng for 14 days. Selection of a 14-day dosing period was based on the principle that it can take up to two weeks for maximal induction of drug metabolizing enzymes involved in the biotransformation of pharmacological compounds. The American ginseng capsules used in our previous studies were made from ground root from a single lot provided by the Ginseng Board of Wisconsin (Wasau, WI). The sample of American ginseng was standardized to ≥5% total ginsenosides, plant steroids of the saponin class that are thought to be the active ingredients of this dietary supplement [8]. The following ginsenoside concentrations were detected by HPLC analysis: Rg1 1.02 mg/g, Re 14.81 mg/g, Rb1 34.28 mg/g, Rc 4.69 mg/g, Rb2 0.65 mg/g, Rb3 0.98 mg/g, and Rd 6.65 mg/g. The sample was also tested for common contaminants including bacteria, fungi, and heavy metals. Ginsenoside plasma concentrations were not measured.

Plasma samples obtained at 4 hours post-dose were utilized for the study since it has been demonstrated that ginsenoside Rb1 concentration peaks approximately 4 h post-dose [9]. HIV RNA-containing plasma was obtained from the Johns Hopkins Pathology plasma bank. One hundred twenty μl of plasma with HIV RNA concentration of 2,177,772 copies/ml was added to 1080 μl test plasma without and with American ginseng. The high HIV RNA concentration was selected to discern between a potential effect of American ginseng on the Cobas assay versus a dilution effect. Matched sample pairs from three volunteers were utilized for the test. We selected three plasma samples of American ginseng based on the assumption that if the assay inhibition was relevant and robust, the effect of American ginseng would have been seen in a few samples with this high dose of American ginseng.

Plasma HIV-1 RNA concentration in these spiked samples was determined using the Roche COBAS Ampliprep/Taqman v2.0 HIV-1 RNA assay with an internal control *(*Limit of Detection 20 copies/ml).

## Results and discussion

In all three plasma pairs (Table 1), the presence of American ginseng had no significant effect on measured plasma HIV-1 RNA concentrations. Log_10_ plasma HIV-1 RNA ranged from 6.02 to 6.09. This variation is negligible and within assay performance limits [4].

**Table 1:** **Effect of American ginseng on plasma HIV RNA measurement**

Sample set	1	2	3			
	- ginseng	+ ginseng	- ginseng	+ ginseng	- ginseng	+ ginseng
**HIV-RNA (copies/ml)**	1109720	1122310	1218900	1165710	1032900	1191440
**HIV RNA (Log**_**10**_ **)**	6.05	6.05	6.09	6.07	6.02	6.08

Roche COBAS Ampliprep/Taqman v2.0 HIV-1 RNA assay (Limit of detection 20 copies/ml).

These results establish that the Roche COBAS Ampliprep/Taqman v2.0 HIV-1 RNA assay does not suffer from interference when used with plasma from subjects receiving American ginseng.

Our study had some limitations: 1) The American ginseng sample tested in our experiments was standardized to ≥5% total ginsenosides and also tested for potential contaminants, as recommended in the literature. Therefore, our results cannot be generalized to other studies that do not follow these recommended standardization procedures; 2) Because ginsenosides or other American ginseng components were not measured in the test samples, we are uncertain about the concentration of American ginseng components in systemic circulation at the time the plasma was sampled. However, previous pharmacokinetic studies [9] have suggested that peak ginsenoside plasma concentrations should occur around the time point we examined; 3) The lack of effect on the COBAS plasma HIV RNA assay described here might not be generalizable to other species, formulations and plant parts of ginseng. Ginseng consists of at least 10 species within the genus *Panax* that are indigenous to Asia (Chinese, Japanese, and Sanchi ginseng) and North America (*Panax quinquefolius*) [8]. There are more than 30 identified ginsenosides, which vary in number and concentration from one species of ginseng to another [8]. These variances in ginsenoside concentrations could potentially lead to a different effect on the performance of HIV RNA assays depending on the species of ginseng being tested. Also, the roots, berries, and leaves of the ginseng plant are known to contain different concentrations of ginsenosides. Since our study used powdered ginseng root, the lack of effect of other parts of the ginseng plant on this viral assay would need to be verified. In addition, difference in ginseng formulations, including dissimilarity in excipient content, is another potential factor that could potentially lead to different effects on the performance of HIV RNA assays. 4) Finally, our experiment tested samples from subjects taking 3000 mg daily of American ginseng for 14 days, which was the highest dose used in one of our previous studies (NCT01136928, http://www.clinicaltrials.gov). The effects of higher doses or treatment duration with American ginseng on viral assays would require further investigation.

## Conclusions

In conclusion, plasma from subjects taking high-dose standardized American ginseng did not interfere with the Roche COBAS Ampliprep/Taqman v2.0 HIV-1 RNA assay performance. These results also suggest that ginseng induced HIV-1 reverse transcriptase inhibition demonstrated *in vitro* [2, 3] is unlikely to be clinically relevant. This finding provides much needed data on the effects of dietary supplements on laboratory tests commonly used in the management of HIV-infected patients, an area of significant concern since the use of herbals and botanicals is common in the HIV-infected community in the United States and in other countries.
